# Q&A: How do plants respond to cytokinins and what is their importance?

**DOI:** 10.1186/s12915-015-0214-5

**Published:** 2015-11-27

**Authors:** Asami Osugi, Hitoshi Sakakibara

**Affiliations:** RIKEN Center for Sustainable Resource Science, 1-7-22, Suehiro, Tsurumi, Yokohama, 230-0045 Japan; Graduate School of Bioagricultural Sciences, Nagoya University, Chikusa, Nagoya, 464-8601 Japan

## Abstract

Cytokinins comprise a family of signaling molecules essential for regulating the growth and development of plants, acting both locally and at a distance. Although much is known about their biosynthesis and transport, important open questions remain.

## What are cytokinins?

**Fig. 1:**
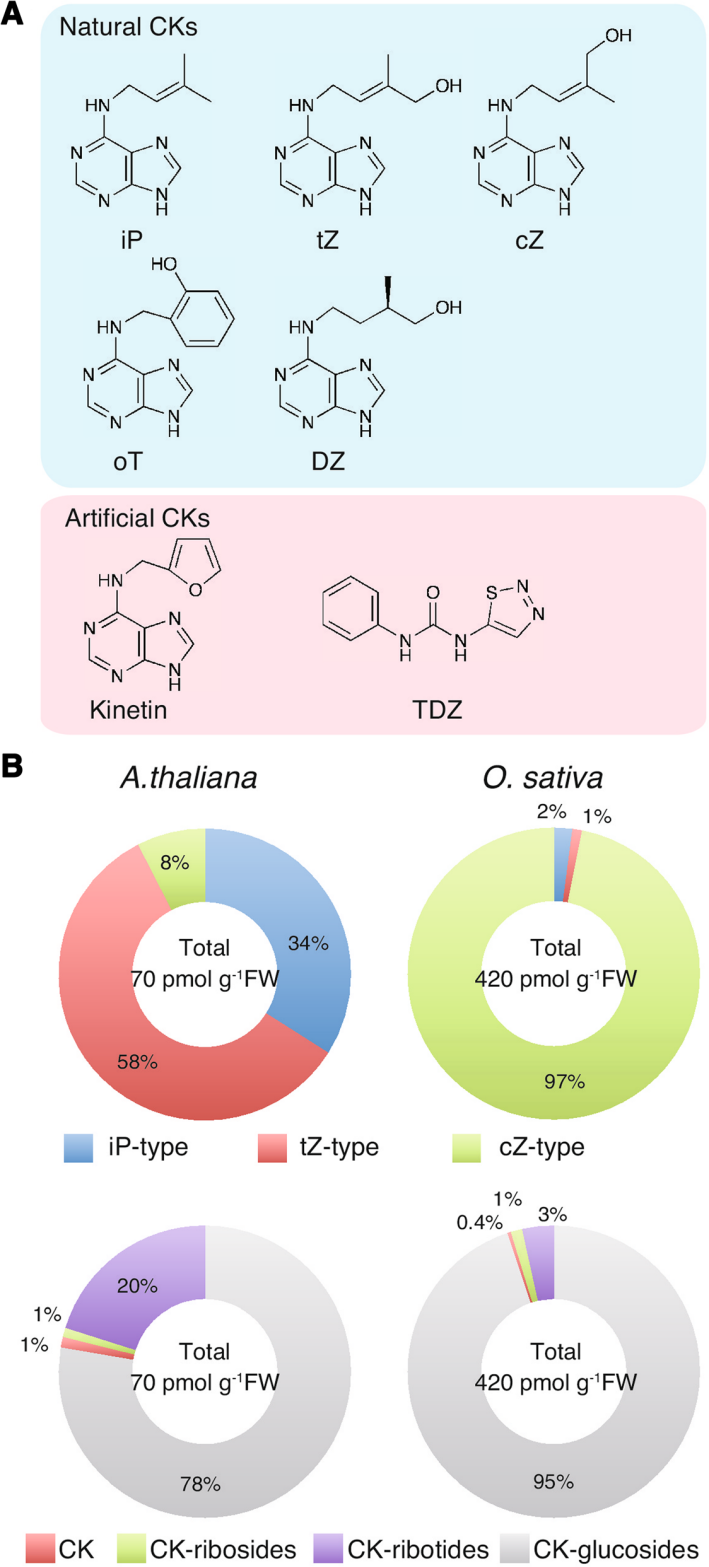
Structure and composition of cytokinins. **a** Structures of various cytokinins (*CKs*). *N* ^6^-(Δ^2^-isopentenyl)adenine (*iP*), *trans-*zeatin (*tZ*), *cis-*zeatin (*cZ*), dihydrozeatin (*DZ*), and *ortho*-topolin (*oT*) are shown as representative natural CKs. Kinetin and thidiazuron (*TDZ*) may activate cytokinin receptors when administered, but are not physiological regulators of plant growth. **b** Pie charts showing relative abundance of cytokinin species in shoots of *Arabidopsis thaliana* (*left*) and *Oryza sativa* (*right*). The *upper charts* show side chain-variant breakdown: tZ and its conjugates (*tZ-type*), iP and its conjugates (*iP-type*), and cZ and its conjugates (*cZ-type*) cytokinins. The *lower charts* show conjugate-variant breakdown: active form (*CK*), ribosides (*CK-ribosides*), ribotides (*CK-ribotides*), and glucosides (*CK-glucosides*). Calculations are based on typical quantification data from [31] and [28] for *Arabidopsis* and rice, respectively. *FW* fresh weight

Cytokinins are a well-studied family of plant hormones. In the 1950s, Skoog and Miller first determined the structural formula of a cytokinin, 6-furfurylaminopurine, a substance isolated from a herring sperm DNA preparation that promotes plant cell division in vitro. It was named kinetin (Fig. 1). However, 6-furfurylaminopurine is artificially formed under specific conditions, such as when DNA preparations degrade or are autoclaved, and was not known to occur naturally. Subsequently, *N*^6^-(Δ^2^-isopentenyl)adenine (iP), *trans-*zeatin (tZ), *cis-*zeatin (cZ), dihydrozeatin, and topolins were identified as natural cytokinins *in planta* (Fig. 1a) [1]. iP and tZ play a major physiological role in *Arabidopsis* and many other plant species due to higher relative abundance and affinity to their receptors. Some plants, including major crops (for example, maize and rice), contain cZ and its conjugates as the most abundant cytokinin species (Fig. 1b). Even in these plants, iP and tZ assume a central role for cytokinin actions, while the physiological significance of cZ has not been fully elucidated. As shown in Fig. 1, cytokinins include various chemicals, but natural cytokinins commonly contain an adenine moiety and a side chain modification at the adenine N6 position. Natural and artificial cytokinins are recognized by common cytokinin receptors [2].

## What are the physiological functions of cytokinins?

Cytokinins were originally defined as chemicals that induce cell proliferation and trigger callus differentiation to shoot when applied with auxins, but now it is known that cytokinins play a key role in many aspects of plant growth and development [3], including embryogenesis, maintenance of root and shoot meristems, and vascular development. They also modulate root elongation, lateral root number, nodule formation, and apical dominance in response to environmental stimuli. Thus, cytokinins are important signaling molecules for regulating growth and development throughout the life of the plant.

## What is the physiological concentration of cytokinins?

The recent development of mass spectrometry technology has enabled us to quantify the concentration of phytohormones and their conjugates at the organ level. Based on such analyses, for which the values are generally 0.1 to 10 pmol g^−1^ fresh weight, it may be estimated that the in vivo concentration of cytokinins is at the nanomolar level, and that concentration may vary between different organs and growth conditions. Notably, if cytokinins are unevenly distributed in the organs at either the tissue or cellular level, their local concentration could be higher or lower. The affinity (apparent *K*_D_) of cytokinin receptors to their ligands is around 1–40 nM [4], which would make the estimated nanomolar concentration of cytokinins physiologically relevant.

## How do plants sense cytokinins?

**Fig. 2:**
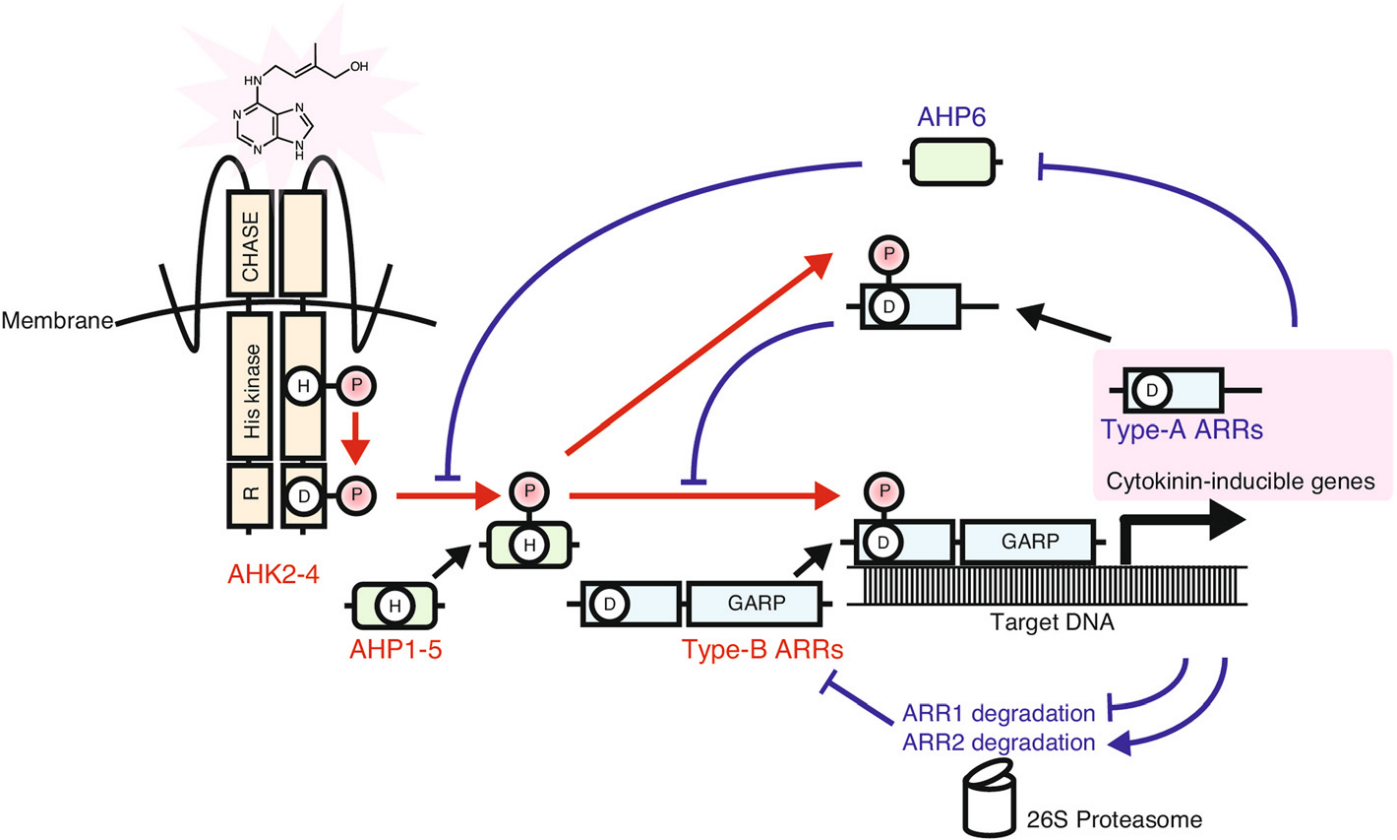
Diagram of the cytokinin two component system (TCS). AHKs (AHK2, AHK3 and AHK4/WOL1/CRE1) are autophosphorylated in response to cytokinins. The phosphoryl group is transferred to type-B ARRs through AHPs. Phosphorylated type-B ARRs bind to target DNA and induce the expression of a set of genes involved in cytokinin primary response. The stability of type-A ARRs, which repress cytokinin TCS signaling, is controlled through proteolysis by the 26S proteasome in a feedback loop. Expression of *AHP6,* which inhibits phosphotransfer between AHKs and canonical AHPs, is repressed by cytokinin. *Red arrows* indicate phosphotransfer. *Blue solid arrows* and *T-end lines* represent positive and negative regulation, respectively

Cytokinins mainly trigger physiological responses through the regulation of gene expression. A two-component system (TCS) is employed to transduce the cytokinin signal to the target genes. The TCS, which typically consists of a sensory histidine kinase and response regulator, was first discovered in prokaryotes, where it serves to enable response to environmental stimuli. Since its first mention in 1996 [5], extensive biochemical and genetic studies have been conducted with *Arabidopsis* to identify and characterize the cytokinin TCS. Today, it is widely accepted that the TCS of cytokinin signaling consists of three groups of proteins in *Arabidopsis*: three histidine kinases (AHKs; AHK2, AHK3 and AHK4/WOL1/CRE1), five histidine-containing phosphotransfer proteins (AHPs; AHP1–AHP5) and eleven type-B response regulators (type-B ARRs; ARR1, ARR2, ARR10–ARR14 and ARR18–ARR21) (Fig. 2) [3].

AHKs are membrane-localized cytokinin receptors which consist of three domains: CHASE, histidine kinase, and receiver [2, 3, 6]. The binding of cytokinins to the AHK CHASE domain triggers autophosphorylation of a His residue in its histidine kinase domain, and the phosphoryl group is internally transferred to an Asp residue in its receiver domain. Recent studies suggest that the cytokinin receptors are localized in both the plasma membrane and the endoplasmic reticulum [7, 8], but it is still unclear whether both are functional or not.

AHPs mediate the transfer of a phosphoryl group from cytosolic AHKs to nuclear-localized type-B ARRs [3, 9]. Type-B ARRs possess a receiver domain for the phosphoryl group, a DNA-binding domain (GARP domain), and a glutamine-rich domain for transcriptional activation [10]. Transcriptional activation by ARRs is repressed in the non-phosphorylated state [10]. When phosphorylated, type-B ARRs can bind target DNA sequences and activate transcription of target genes.

Since each member of the AHK, AHP and type-B ARR gene families is functionally redundant, clear phenotypic differences are not observed in their single mutants. However, responsiveness to cytokinins is severely reduced and growth phenotypes could be observed in multiple mutants within each gene family [11–13], indicating that the cytokinin TCS plays a central role in cytokinin responses in plants.

## How does the TCS modulate its signaling flux?

In order to finely regulate cytokinin signaling, multiple feedback loops of the cytokinin TCS are employed to ensure the appropriate signaling flux (Fig. 2). In addition to the 11 type-B ARRs, the *Arabidopsis* genome has ten type-A ARR genes (*ARR3*–*ARR9*, *ARR15*–*ARR17*) [3], and some of them are direct targets of type-B ARRs. Like type-B ARRs, type-A ARRs possess a receiver domain and receive a phosphoryl group from AHPs, but they lack a DNA-binding domain. Thus, type-A ARRs can potentially inhibit the cytokinin signaling flux by competing with type-B ARRs for phosphate transfer [14].

Differential regulation of posttranslational stability of type-B ARRs is involved in the modulation of the TCS signaling flux. Cytokinins promote degradation of ARR2 via the 26S proteasome [15] while they stabilize ARR1 by preventing degradation by 26S proteasome [16]. On the other hand, KISS ME DEADLY proteins (KMDs), a family of F-box proteins, are involved in degrading ARR1, ARR2 and ARR12, but the detailed mechanisms have yet to be discovered [17].

It has also been reported that an AHP homologue, AHP6, which lacks the conserved histidine for phosphoryl group transfer*,* physically interacts with AHKs but does not receive a phosphate, suggesting that AHP6 inhibits cytokinin response through competition with canonical AHPs. Cytokinins repress *AHP6* expression [18], which suggests that the promotion of cytokinin signaling flux is in part mediated by the down-regulation of *AHP6*. It was reported that cell-specific expression of *AHP6* serves spatial specification of cytokinin signaling [19, 20]. In addition, cytokinins induce the expression of a cytokinin receptor gene, *AHK4*/*WOL1*/*CRE1*, which might lead to increased sensitivity to cytokinins [21]. It has been proposed that occurrence of these feedback loops in an organ- or a cell-specific manner is important for regulation of cytokinin signaling flux, as described later.

## How are cytokinins produced and metabolized in plants?

**Fig. 3:**
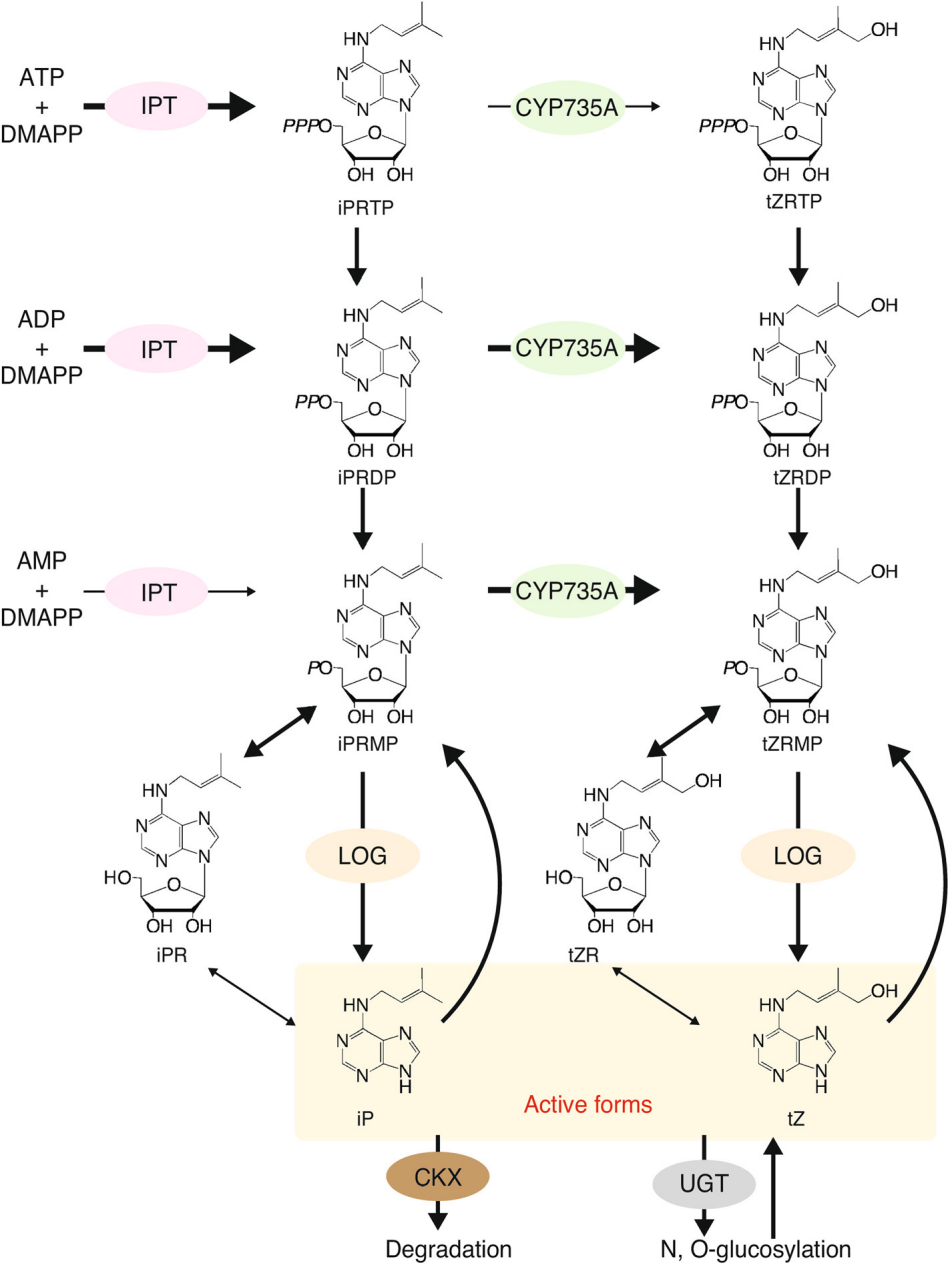
Current model of iP and tZ biosynthesis and metabolic pathways in *Arabidopsis*. In *Arabidopsis*, IPT preferentially utilizes ATP and ADP, and CYP735A preferentially utilizes iPRMP and iPRDP, as substrates. LOG exclusively reacts with their monophosphate forms. Active cytokinins are degraded by CKX, glucosylated by UGT, or reverted to their precursors by the purine salvage pathway

Cytokinin levels in vivo are determined by the balance between biosynthesis and catabolism. Biosynthesis of cytokinins is regulated by three key enzymes (Fig. 3). First, adenosine-phosphate isopentenyltransferase (IPT) catalyzes the formation of iP ribonucleotides from dimethylallyl diphosphate (DMAPP) and adenine nucleotides, with preferential use of ATP or ADP [22, 23]. Cytochrome P450 monooxygenase 735A (CYP735A) hydroxylates the end of the prenyl side chain of iP ribonucleotides, with a preference for iP-riboside 5’-monophosphate (iPRMP) or diphosphate (iPRDP), to produce tZ ribotides [24]. Finally, LONELY GUY (LOG), a phosphoribohydrolase, converts the precursors iPRMP and tZ-riboside 5’-monophosphate (tZRMP) to their active forms, iP and tZ, respectively [25]. Although cytokinin ribonucleosides, such as iP ribonucleoside and tZ ribonucleoside, are widely found in plant tissues, they are considered to be precursors and mainly converted to their active form via LOG after phosphorylation, because *log* septuple mutants, in which the LOG-dependent pathway is impaired, exhibit severe cytokinin-deficient phenotypes in *Arabidopsis* [26]. The biosynthesis of cytokinins is regulated through *IPT* genes by various internal and external environmental stimuli, such as nitrogen sources [27, 28], and other phytohormones [29].

For the catabolism of cytokinins, cytokinin oxidase/dehydrogenase (CKX) irreversibly degrades cytokinins by cleaving the unsaturated isoprenoid side chain, which results in the formation of adenine and the corresponding aldehyde [3]. Glycosylation of cytokinins also plays an important role in modulating cytokinin activity: *N*-glucosylation of the N7 and N9 positions of the adenine moiety and *O*-glucosylation of the zeatin side chain are catalyzed by UGT76C1 and C2, and 85A1, respectively, in *Arabidopsis* [3, 30]. Because of their biological stability, the glucosides are the most abundant components of cytokinin derivatives, making up 80 % or more of total cytokinin-related compounds in plants (Fig. 1b). These glucosides are thought to be sequestered in the vacuole. In addition, ribosidation and ribotidation by purine salvage pathway enzymes contribute to the cycling of cytokinins, through reverting active forms back to inactive precursors (Fig. 3). Thus, homeostasis of cytokinin activity is maintained by multiple metabolic systems.

## Where are cytokinins produced in plants?

**Fig. 4:**
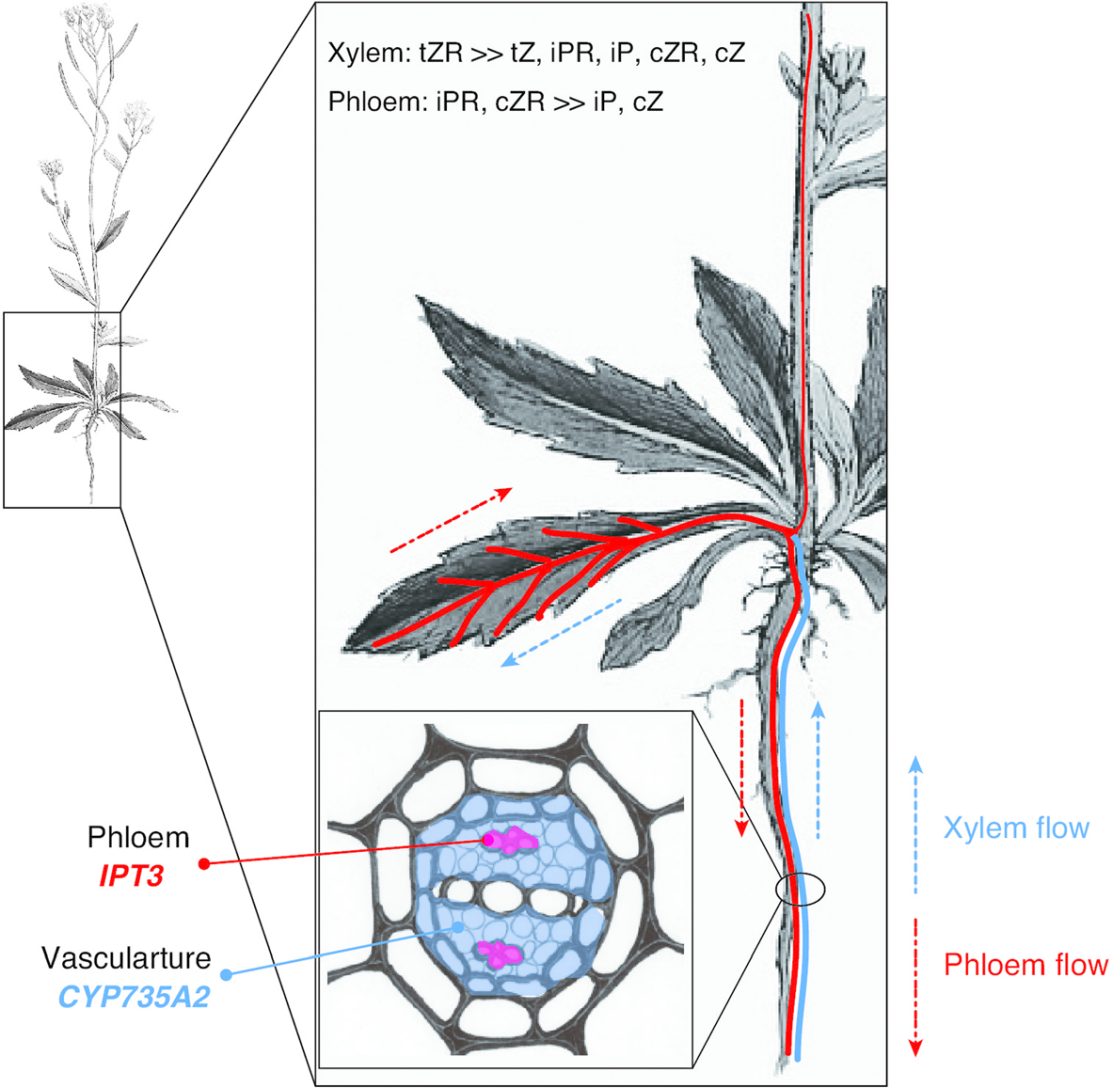
Spatial expression patterns of *IPT3* and *CYP735A2* in *Arabidopsis*. Spatial expression patterns of *IPT3* and *CYP735A2* are indicated in *red* and *blue*, respectively. *IPT3* is predominantly expressed in phloem, and *CYP735A2* in root vasculature. tZR is the major form of xylem cytokinins, and iPR and cZR are found in phloem [40], suggesting that iP-type and tZ-type cytokinins are directionally translocated between organs. The *Arabidopsis* picture is modified from Sowerby et al. [53]

Cytokinins are more abundant in developing tissues and organs, such as root tip, shoot apex, cambium, and immature organs, and initially it was thought that cytokinins are synthesized in these limited tissues and organs. However, recent studies provide us with insight into cytokinin-producing and -degrading sites. There are seven *IPT* genes (*IPT1* and *IPT3*–*IPT8*), two *CYP735A* genes (*CYP735A1* and *A2*), seven functional *LOG* genes (*LOG1*–*LOG5*, *LOG7* and *LOG8*) and seven *CKX* genes (*AtCKX1*–*AtCKX7*) in *Arabidopsis* [3]. These gene families show various expression patterns [27, 29, 31–33]. Notably, *IPT3* is expressed in the phloem in both roots and aerial organs, suggesting that the precursor of iP-type cytokinins could be synthesized in a wide range of plant parts [27, 29] (Fig. 4). On the other hand, *CYP735A* is predominantly expressed in root vasculature [31]. Such spatial distribution of *IPT* gene and *CYP735A* expression causes the preferential synthesis of tZ in roots and of iP in shoots. Expression of the *LOG* gene family covers almost all organs. Thus, the activation step of cytokinin synthesis can occur wherever the *LOG* genes are expressed [32]. These expression patterns suggest that cytokinins play roles in both long-distance and local signaling.

## How important are locally produced cytokinins for plant development?

**Fig. 5:**
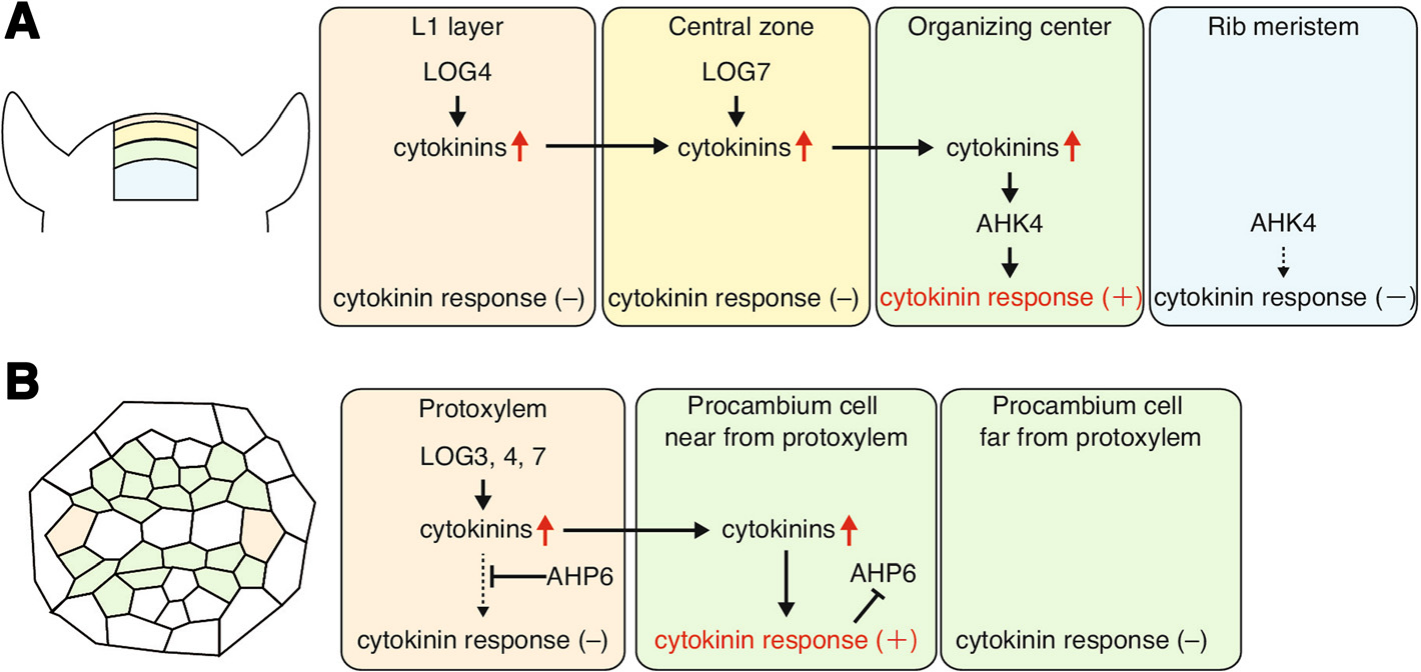
Local function of cytokinins in shoot apical meristem and root tip. **a** Schematic diagram of cytokinin production and response in shoot apical meristem. Active cytokinins are produced in the epidermal L1 layer and central zone. They diffuse basipetally and function in the *AHK4*-expressing organizing center. **b** Schematic diagram of cytokinin production and response in root tip. Active cytokinins are produced in *AHP6*-expressing protoxylem. They diffuse and function in adjacent procambium cells while repressing *AHP6* expression

The importance of cytokinins’ local action is best characterized by their role in maintenance of shoot apical meristem and root vascular development (Fig. 5). In *Oryza sativa* (rice), a single *log* mutation confers reduced shoot apical meristem size and abnormal floral organ development, although ten *LOG-like* genes are expressed in various organs [25]. Similarly, although the genes of the *Arabidopsis LOG* family are expressed in various types of tissues and organs and thought to be functionally redundant [32], the single *log7* mutation causes reduction of shoot apical meristem size [26]. Rice *LOG* and *Arabidopsis LOG7* are expressed in a restricted region of the upper part of the shoot apical meristem. Thus, loss of function of certain *LOG* genes expressed in this specific site is not fully complemented by other *LOG* genes expressed in other parts of the plant. These facts indicate that maintenance of shoot apical meristem activity requires site-specific activation of cytokinins.

In order to maintain shoot apical meristem, it is necessary to channel the cytokinin signal towards the organizing center [34]. In shoot apical meristem, active cytokinins are produced in the L1 layer by *LOG4* and in the central zone by *LOG7* [35, 36]. The organizing center and rib meristem are responsive to cytokinins via *AHK4*/*WOL1*/*CRE1* (Fig. 5a) [36]. Endogenous cytokinins activate cytokinin signaling only in the organizing center but not in the rib meristem, although exogenously applied cytokinins activate both of these tissues (Fig. 5a) [36]. This indicates that the longitudinal diffusion of cytokinins to adjacent regions and local action in the restricted cells are important for proper meristem function.

The importance of local activation of cytokinins is also shown in the early stage of root vascular development. In root tip vasculature, which consists of procambium, protoxylem, metaxylem and phloem (Fig. 5b), cytokinin-activating *LOG3* and *LOG4* are specifically expressed in protoxylem cells, while cytokinin-perceiving *AHK4*/*WOL1*/*CRE1* is expressed in the procambium [19, 20]. Analysis using marker genes for cytokinin response revealed that procambium cells adjacent to protoxylem respond to cytokinins more strongly than distant procambium cells [19, 20], indicating that local activation of cytokinins in protoxylem specifies cytokinin perception cells in procambium. Since cytokinins inhibit procambium-to-protoxylem differentiation, this regulation may be necessary for proper positioning of those cells during vascular development.

Root morphogenesis is abnormal in the *log3 log4 log7* triple mutant, possibly due to reduced levels of active cytokinin [19]. Expression of *LOG3* under a xylem precursor cell-specific promoter rescues this phenotype. However, ectopic expression of *LOG3* in the phloem, through which cytokinins are systemically transported, does not. Therefore, it is suggested that the locally synthesized cytokinins are necessary for root morphogenesis.

## How important are distantly translocated cytokinins for plant growth and development?

Movement of cytokinins between organs has been shown by tracer experiments using isotope-labeled cytokinins [31, 37–39] and detection of cytokinins in vascular saps also supports translocation of cytokinins [40]. In general, long-distance signals are translocated through xylem and phloem, two major conduits for material transfer in plant vasculature. Intriguingly, these studies show that cytokinin species are unevenly distributed: tZ-type cytokinins are more abundant than iP-type in xylem sap and vice versa in phloem sap [40] (Fig. 4). These studies imply that iP-type and tZ-type cytokinins are directionally translocated and transmit different biological messages between organs.

Grafting experiments support the importance of cytokinins as long-distance signals. The *Arabidopsis ipt1 ipt3 ipt5 ipt7* quadruple mutant shows severely reduced cytokinin content and shoot and root growth-deficient phenotypes [41]. Reciprocal grafting between the quadruple mutant and wild type rescued the growth-deficient phenotypes in shoot and root with accompanying recovery of cytokinin content [41], indicating a role of root-borne cytokinins in shoot and vice versa.

Grafting experiments also show the importance of root-borne tZ for normal shoot growth. In the *cyp735a1 cyp735a2* double mutant, tZ-type cytokinin content is severely reduced without affecting total cytokinin quantity, and shoot growth is retarded [31]. When the shoot of the double mutant is grafted onto wild-type stock, the shoot phenotype is complemented with accompanying recovery of tZ-type cytokinin content, suggesting that side chain modulation of cytokinins has a specialized role in their long-distance translocation for shoot growth regulation.

As for the role of shoot-borne cytokinin in roots, impairing phloem transport destabilizes root vascular development with an accompanying reduction of basipetal cytokinin translocation in *Arabidopsis* [37]. Thus, shoot-borne cytokinins, via phloem, could participate in normal development of root vasculature in coordination with locally produced cytokinins.

In *Lotus japonicas*, root nodule number is reduced in the *Ljipt3* cytokinin biosynthesis gene mutant [38], as well as in a *Ljipt3* knockdown transgenic line [42]. Grafting between *Ljipt3* shoot and wild-type root stock represses nodulation, while reverse grafting does not [38]. This suggests that shoot-derived cytokinins function as a repression signal of nodulation. It is notable that cytokinins synthesized in the root are not significantly involved in regulation of nodule number, although the mechanism through which the origin of cytokinins is determined remains to be elucidated.

So far, studies have shown that both xylem and phloem sap contain vast amounts of cytokinin ribonucleosides [40], suggesting that cytokinin ribonucleosides are translocated through the vasculature. It is believed that the cytokinin ribonucleosides are converted to their ribonucleotides followed by activation via LOG where they function.

## How is long-distance translocation of cytokinins regulated?

An elaborate translocation system is necessary for the regulation of organ-to-organ communication via cytokinins. Recent studies identified *ATP-binding cassette transporter subfamily G14* (*ABCG14*) as a key gene for appropriate root-to-shoot cytokinin translocation [39, 43]. In *abcg14*, tZ-type cytokinin contents are greatly reduced in xylem sap, and the dwarf phenotype of *abcg14* is rescued in grafted plants between *abcg14* shoot and wild-type root stock [43], indicating that *ABCG14* is an essential gene for root-to-shoot translocation of cytokinins. Since the biochemical properties of ABCG14 have not been well characterized, the substrate of ABCG14 has not been identified. In addition to ABCG14, *purine permease 1* and *2* (*PUP1* and *PUP2*) and *equilibrate nucleoside transporter* (*ENT*) have been shown in in vitro studies to be involved in transport of cytokinins [44, 45]. However, their functions *in planta*, especially in long-distance translocation of cytokinins, are still poorly characterized.

## What determines the site of cytokinin action?

Spatial regulation of LOG expression is one of the determining factors that specify the functional sites of cytokinins, and expression of each *LOG* family gene is regulated in a site-specific manner [25, 32]. Recently, a mechanism for this site-specific expression of *LOG* genes has been reported in flower development in *Arabidopsis* [46]. In a mutant of *APETALA1* (*AP1*), a MADS-BOX transcriptional factor, abnormal floral organs are observed. *AP1* directly represses *LOG1* expression, and possibly in sepals where their expression overlaps. *LOG1* repression, under control of the *AP1* promoter, partially rescued the *ap1* phenotype, indicating that sepal-specific *LOG1* repression is required for normal flower development. In addition, expression of a *LOG* homolog is directly activated by *KNOTTED1* (*KN1*) in maize [47]. It is thought that *KN1* might provide site-specific regulation of the *LOG* homolog’s coordination with certain *BEL1-LIKE HOMEOBOX* (*BLH*) gene products, which interact with KN1 to bind DNA.

Recent studies have also revealed the importance of cytokinin oxidase/dehydrogenase (CKX) function as a metabolic attenuator of local cytokinin action. As well as *LOG*, the expression of each *CKX* family gene is regulated in a site-specific manner [33]. Loss-of-function mutants of specific *CKX* genes in rice (*OsCKX2*) and *Arabidopsis* (*CKX3* and *CKX5*), which are expressed in reproductive meristem, cause increased cytokinin levels, leading to larger meristem size and an increase in reproductive organ number [48, 49], clearly showing that the CKXs fine-tune active cytokinin levels at the expression site. *CKX3* expression in the organizing center and *CKX5* broadly in the meristematic domain [49] may be particularly important for ensuring the site-specific cytokinin response (Fig. 5a). It is also intriguing that *Arabidopsis* AP1, which negatively regulates *LOG1* expression, positively regulates *CKX3* in the sepal [46]. This regulation is important for development of a determinate floral meristem.

Another candidate to control functionality of cytokinins is the family of AHK proteins. AHK promoter:reporter gene analyses revealed that the cytokinin receptors are expressed in various tissues in the plant, but that *AHK3* is preferentially expressed in aerial organs, such as rosette leaves, *AHK4*/*WOL1*/*CRE1* is expressed in root, and *AHK2* is expressed in both [11]. Interestingly, it is reported that their affinities to ligands are different: *AHK2* and *AHK4*/*WOL1*/*CRE1* bind with similar affinity to tZ as well as iP, while *AHK3* has less affinity to iP than to tZ [4, 50]. Thus, it is expected that tZ plays a major role as a cytokinin in shoots. This supports previous work with *cyp735a1 cyp735a2* identifying a specialized function of tZ in shoot development [31].

Some other components of the cytokinin TCS also contribute to determining the sites of action of cytokinins. Cytokinins induce expression of *WUSCHEL* (*WUS*), a transcriptional factor expressed in the organizing center of the shoot apical meristem. WUS directly binds to the promoters of *ARR5*, *ARR6*, *ARR7,* and *ARR15*, and represses their expression (Fig. 5) [34]. This regulation establishes a local spatial domain for the organization of a stem cell niche in the shoot apex. (Fig. 5) [51].

## What interactions occur between cytokinins and other phytohormones?

Signaling systems of phytohormones build a network and mutually regulate signaling, transport, and metabolic systems. Interplay between cytokinins and auxin is one of the best-characterized cases of hormone–hormone interaction. Recent studies shed light on the importance of cytokinin–auxin interaction for auxin traffic and specification of cytokinin action sites in root vascular development. Cytokinin signaling in the procambium up-regulates expression of PINs, a family of auxin efflux carriers, and promotes their distribution in the plasma membrane from anticlinal to periclinal [52]. The bisymmetric distribution of PINs channels basipetally translocates auxin to protoxylem via the procambium. The auxin is perceived in the protoxylem and induces *LONESOME HIGHWAY* (*LHW*) and *TARGET OF MONOPTEROS5* (*TMO5*). They directly induce *LOG3* and *LOG4*, which activate cytokinins, and *AHP6*, which inhibits cytokinin responses in the protoxylem [19, 20]. Thus, the auxin-induced LOG3 and LOG4 in protoxylem provides cytokinins to adjacent procambium for proper root vascular development while the induced AHP6 inhibits cytokinin response (Fig. 5b) [19, 20]. Consequently, the procambial cytokinin response regulates PIN expression and distribution.

## What is the important question for the future?

Cytokinins positively regulate agriculturally important traits such as grain size and biomass [3] but they also promote unfavorable phenotypes such as inhibition of root elongation. Therefore, spatio-temporal regulation of cytokinins is required for appropriate function in specific organs. In this article, we have discussed the spatial regulation of cytokinin biosynthesis and the role of these hormones in signaling. In order to better utilize cytokinin action to enhance beneficial traits of crops, a deeper understanding of their temporal regulation will be necessary.
